# Global Priorities for Marine Biodiversity Conservation

**DOI:** 10.1371/journal.pone.0082898

**Published:** 2014-01-08

**Authors:** Elizabeth R. Selig, Will R. Turner, Sebastian Troëng, Bryan P. Wallace, Benjamin S. Halpern, Kristin Kaschner, Ben G. Lascelles, Kent E. Carpenter, Russell A. Mittermeier

**Affiliations:** 1 Betty and Gordon Moore Center for Science and Oceans, Conservation International, Arlington, Virginia, United States of America; 2 Department of Biology, Lund University, Lund, Sweden; 3 Oceanic Society, Ross, California, United States of America; 4 Nicholas School of the Environment, Duke University Marine Laboratory, Beaufort, North Carolina, United States of America; 5 National Center for Ecological Analysis and Synthesis, Santa Barbara, California, United States of America; 6 Bren School of Environmental Science & Management, University of California Santa Barbara, Santa Barbara, California, United States of America; 7 Imperial College London, Ascot, United Kingdom; 8 Department of Biometry and Environmental System Analysis, Albert-Ludwigs-University, Freiburg, Germany; 9 Centre de Synthèse et d'Analyse sur la Biodiversité (CESAB), Aix-en-Provence, France; 10 Global Seabird Programme, Birdlife International, Cambridge, United Kingdom; 11 Department of Biological Sciences, Old Dominion University, Norfolk, Virginia, United States of America; 12 Global Species Programme, International Union for the Conservation of Nature, Gland, Switzerland; 13 Conservation International, Arlington, Virginia, United States of America; New England Aquarium, United States of America

## Abstract

In recent decades, many marine populations have experienced major declines in abundance, but we still know little about where management interventions may help protect the highest levels of marine biodiversity. We used modeled spatial distribution data for nearly 12,500 species to quantify global patterns of species richness and two measures of endemism. By combining these data with spatial information on cumulative human impacts, we identified priority areas where marine biodiversity is most and least impacted by human activities, both within Exclusive Economic Zones (EEZs) and Areas Beyond National Jurisdiction (ABNJ). Our analyses highlighted places that are both accepted priorities for marine conservation like the Coral Triangle, as well as less well-known locations in the southwest Indian Ocean, western Pacific Ocean, Arctic and Antarctic Oceans, and within semi-enclosed seas like the Mediterranean and Baltic Seas. Within highly impacted priority areas, climate and fishing were the biggest stressors. Although new priorities may arise as we continue to improve marine species range datasets, results from this work are an essential first step in guiding limited resources to regions where investment could best sustain marine biodiversity.

## Introduction

Widespread impacts of human activities on the oceans [1] continue to cause declines in species diversity and abundance [2], [3]. As recognition of the benefits that healthy marine ecosystems provide to people increases [4], [5], protecting biodiversity and the essential ecosystem services it supports has become a priority for the scientific community, resource managers, and national and international policy agreements, including the Convention on Biological Diversity (CBD) [6]. Decreases in species richness or abundance can threaten ecosystem services such as fisheries or nutrient cycling, and can reduce overall ecosystem stability and resilience [7], [8]. These declines have been documented for numerous marine ecosystems [9], and can sometimes lead to major shifts in food web dynamics [10]–[12]. Many of these changes can be attributed to human impacts such as climate change, overfishing, and pollution [1], [13], [14]. However, limited capacity and financial support for conservation and management necessitate that resources be directed to regions where investment could best sustain areas of high marine biodiversity and their associated ecosystem services [15], [16].

Identification of priority areas such as ‘hotspots’ [16], high-biodiversity wilderness areas [17], or other categorizations such as ecoregions [18] have been essential tools for conservation planning in terrestrial and marine ecosystems [15]. However, identifying spatially explicit areas of high biodiversity associated with either high or low human impact for marine ecosystems has never been done, despite their utility in terrestrial conservation [15]. We combined the most extensive global compilation of species distribution data currently available with high-resolution data on human impacts to identify emergent patterns as a key input to achieving global marine biodiversity conservation objectives.

Many biological and socioeconomic measures are potentially important for determining places of high conservation value, but relative levels of biodiversity and human impact are among important considerations for conservation prioritization efforts [19]–[21]. We focused our analyses on two fundamental metrics of biodiversity: species richness and species endemism [19], [20], which are not necessarily spatially concordant [22]. These two metrics of diversity are thought to be important for different reasons. Dynamics can vary by ecosystem and species [23], but marine communities with greater species richness can have greater resilience to environmental stress than similar communities with lower species richness [24]. Endemic or range-restricted species are generally considered to be at greater extinction risk due to localized human or natural disturbances [15]. Conserving places with high endemism is critical for preventing biodiversity loss and maintaining genetic variability [15], [16]. Although previous efforts [25] have analyzed peaks in marine species richness for select taxa, we used finer-scale data and included two metrics of endemism to create spatially explicit maps of diversity classified by degree of impact. Many conservation efforts require data at these finer scales for planning purposes [21].

There are two broad approaches to identifying places that may be important because of the endemic species they harbor. One approach, hereafter ‘range rarity’, identifies the greatest concentrations of relatively rare or range-restricted species. This approach has been used previously to identify conservation priority areas in terrestrial and marine ecosystems [15], [20], [26]–[28]. The other approach is to identify those places that have species with restricted ranges, independent of the number of species present. Because this approach divides range rarity values by species richness, we hereafter refer to it as ‘proportional range rarity’. By including both metrics of endemism, we can provide complementary insights into the places that may be important for protecting endemic species. We identified locations where species richness and these two metrics of endemism peaked across taxonomic groups and coupled these results with a high resolution model of estimated cumulative human impacts [1] to generate a spatially explicit roadmap for prioritizing particular places and types of impacts for marine conservation action.

## Methods

### Species and human impact data

We used modeled species distribution data for 12,497 species from several sources [29]–[31] to get the greatest taxonomic coverage as possible. The overall species database covered more than 21 phyla from 966 families (Table S1). For ∼90% of the species in our analyses, range maps were derived from AquaMaps [30], an online species distribution modeling tool that produces standardized, digital range maps of aquatic species (www.aquamaps.org) (Table S1). Although modeled species distribution databases can contain inaccuracies [32], they represent the most comprehensive and highest resolution biodiversity distribution data available [30] for this purpose. AquaMaps maps are based on an environmental niche envelope model and species-specific habitat usage derived from occurrence records available through the Global Biodiversity Information Facility (www.gbif.org), supplemented with expert knowledge and additional information obtained through online species databases such as FishBase (www.fishbase.org) and SeaLifeBase (www.Sealifebase.org). These maps predict relative probabilities of species occurrence (ranging from 0.00–1.00) at a resolution of 0.5 degree latitude-by-longitude cells. For this analysis, we applied a probability threshold value of 0.00 or greater for a cell to be considered within a species' distribution, which is the least conservative estimate of a species range, but is most comparable to the polygon maps of maximum range extents used for other species mapping exercises (e.g., those of Birdlife International and the International Union for the Conservation of Nature - Global Marine Species Assessment) [29], [33]. We describe sensitivity analyses and results of this assumption below.

Across all species distributions, we calculated species richness and both metrics of endemism within a global grid of 2,591.4 km^2^ icosahedral Snyder equal area hexagons (*n* = 197,316). We assigned species to hexagons based on the overlap between the raster grid (i.e. species from AquaMaps) or the polygon data (i.e. corals and seabirds) and the hexagon grid. In other words, if a species range overlapped at all with a hexagon, it was scored as present. However, in cases where only a portion of the hexagon was considered to be part of a species range, we weighted values for richness, range rarity, or proportional range rarity in that cell by the percentage of the hexagon that the species range occupied. No minimum range area was set for inclusion as it sometimes is for terrestrial systems to avoid biasing results towards very restricted range species. In the datasets we used, species ranges were not restricted to such a small area that they would bias results. We defined species richness as the number of species within each hexagon (Fig. S1A). We quantified endemism using two approaches. *Range rarity* [34] was defined as:
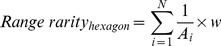
where for each species *i* of *N* species per hexagon, 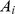 is the total range area for that species *i* including all areas inside and outside of the hexagon and 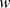 is the fraction of the hexagon that overlaps with the species range (Fig. S1B; i.e., *w* = 1 if the species range covered the whole hexagon). Range rarity reflects both the number of species and the size of their ranges, which is a common way to delineate priorities based on endemism because it quantifies the number of relatively restricted range species within a cell [20], [26]. To calculate *proportional range rarity*, we used the same formulation of range rarity, but then divided the values by richness to remove its confounding effect (Fig. S1C). For analytical purposes, range rarity values were then multiplied by 100,000 and proportional range rarity values were multiplied by 1,000 to create integer datasets.

For all metrics of biodiversity (richness, rarity, and proportional range rarity), we used a standard area-based measure for identifying places with the greatest diversity. For each metric, we identified the 5% of grid cells within the total area of exclusive economic zones (EEZs) that had the highest values (equating to 8,107,940 km^2^ of global EEZ area) and, separately, the 5% of grid cells in areas beyond national jurisdiction (ABNJ) that had the highest values (equating to 11,490,800 km^2^ of global ABNJ area). For example, for species richness in EEZs, we selected those cells with the highest richness values that summed to 5% of the total global EEZ area. The use of an area threshold rather than a value threshold meant that each diversity metric contributed an equal area to the assignment of priorities. We selected a 5% threshold because it has been used previously in terrestrial analyses [21] and was specific enough to ensure that we could separate very high diversity areas within the coastal zone, but broad enough to enable the identification of multiple candidate areas in different regions.

To assess the degree of human impact on high biodiversity areas, we used estimated spatially explicit cumulative impact data (Fig. S1D) from Halpern *et al.* 2008 [1]. This model includes 17 different drivers of change within marine ecosystems, weighted by the sensitivity of the ecosystems present in a grid cell to each of these drivers [1]. The native resolution of these data is 1 km^2^. We again used an area-based approach, this time with a 10% area threshold, to identify the highest and lowest impact areas within EEZs and ABNJ. We defined areas of high impact as the 10% of total EEZ area having the highest impact values or, separately, the 10% of total ABNJ area having the highest impact values. Similarly, we defined low impact areas as the 10% of EEZ (or ABNJ) area having the lowest impact values. We included both high impact and low impact areas because alternative conservation approaches advocate ‘reactive’ protection of critical, yet highly impacted places [15], [20] as well as more ‘proactive’ protection of important wilderness areas.

We used a 10% area threshold for impact values for two reasons: 1) preliminary analyses indicated a 5% area threshold to be too restrictive in terms of the area that would have potential overlap with the top 5% of EEZ or ABNJ area by richness, range rarity, or proportional range rarity, and thus was not informative, and 2) management interventions can address a broader degree of impact levels rather than just the very most or least impacted. For some analyses, we grouped the 17 drivers of ecological change into four broad categories: climate (temperature, acidification and ultraviolet radiation), fishing (pelagic low bycatch, pelagic high bycatch, demersal low bycatch, demersal high bycatch, demersal habitat modifying, and artisanal fishing), land-based (nonpoint inorganic, nonpoint organic, nutrient, and direct human), and ocean-based pollution (benthic structures, shipping, ocean-based pollution, and species invasions), following Halpern *et al.*2008 [1].

### Spatial concordance of biodiversity and estimated human impacts

**Figure 1:**
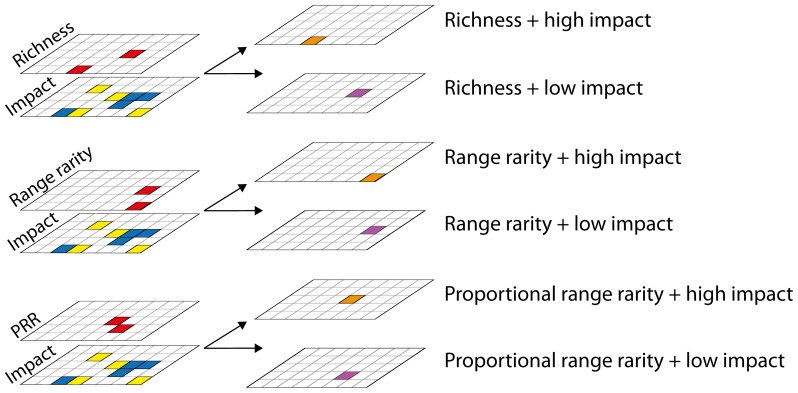
Analytical process for identifying priority areas according to their biodiversity and impact levels. This illustration shows the process for identifying priority areas, which was done separately for EEZ and ABNJ areas. For each metric of diversity—richness, range rarity and proportional range rarity—we identified the top values within 5% of total EEZ or ABNJ area (red). We also identified the top (yellow) and bottom (blue) 10% of EEZ and ABNJ area by impact. Priority areas were then identified by the area of overlap between each biodiversity metric and areas of high impact (orange) or low impact (purple).

We developed 6 classifications of marine spatial priorities based on our classifications of 3 different measures of biodiversity (richness, range rarity, and proportional range rarity) at 2 different levels of human impact (low and high; Fig. 1). To determine the spatial overlap between impact and diversity, we first downscaled the species data to match the resolution of the human impact data (1 km^2^). Although the native resolution of the species data is considerably coarser than the impact data, we wanted to preserve important spatial variability within the human impact data for our analyses. For example, many fishing, nutrient and sedimentation impacts are concentrated within narrow continental shelf areas [1]. We chose not to upscale the human impact data, as doing so would have artificially spread values from narrow and highly impacted shelf areas to less-impacted waters nearby. We did not use any smoothing algorithm in the downscaling process so patterns in the data remained true to the coarseness of the data involved.

We then determined where grid cells with the highest richness, range rarity, or proportional range rarity values overlapped with the highest or lowest impact values. This analysis resulted in 6 grids that designated areas of high biodiversity and high or low impact within EEZ areas and 6 separate, additional grids for ABNJ areas (Fig. 1). To identify concentrations of high biodiversity and high or low impact, we converted the 12 raster grids (6 each for EEZ and ABNJ) to points and calculated 3×3 point density functions with ArcGIS at 0.25 degree. Running the point density function allowed us to identify high-density clusters of candidate priorities and connect areas in the interstices into contiguous areas to create more broadly defined priority areas. For each of the 12 grids, we included high-density areas that amounted to 1% of total EEZ or 1% of total ABNJ area. If none of the 6 grids overlapped, priority areas would equal exactly 6% of total EEZ or ABNJ area. Because the different grids do overlap, the actual percentage of EEZ or ABNJ area identified is approximately 5% of EEZ or ABNJ area, respectively. We aimed for an approximate target of 5% of EEZ or ABNJ area because these areas were specific enough to identify areas of very high biodiversity and either low or high impact. A 5% target also leaves room to achieve CBD targets [6] based on other important criteria related to socioeconomic, governance, or other biodiversity considerations. Current CBD targets are to conserve 10% of coastal and marine areas by 2020. To explore the outcome of an alternative threshold, we also calculated where diversity was greatest using a 10% area threshold for the biodiversity metrics and using the same the impact thresholds (Fig. S3).

### Validations and sensitivity analyses

Nearly every species range distribution dataset, including those based on point data, has to be modeled in some respect to recreate distributions. Even for marine mammals, which are one of the most heavily sampled taxa, point occurrence records are currently only available for <60% of known marine mammals, and 70% of all available sighting records come from continental shelf waters of the Northern Hemisphere, according to the Ocean Biogeographic Information System [35]. In order to validate the general diversity patterns in our dataset, we conducted a linear regression between our normalized species richness values and the normalized species richness values of Tittensor *et al.* 2010 [25]. The Tittensor *et al.* 2010 dataset focuses on a somewhat different suite of species and uses different procedures for generating species range maps from point observations [25].

In the marine realm, where there are many wide-ranging and cosmopolitan species, patterns of biodiversity derived from maximum range maps like those used here may overestimate presence on continental shelf areas. These presence/absence range maps implicitly assume homogeneous species occurrence and thus can overestimate the relative importance of shelf and slope habitat for truly oceanic species. Therefore, we conducted sensitivity analyses to examine how priorities for richness, range rarity, and proportional range rarity would change if more conservative presence thresholds of probabilities >0.40 or >0.80 were used rather than the ≥0.00 threshold used in the main analyses. A higher probability of occurrence restricts species presence to species-specific areas of high environmental suitability, which corresponds to what may be considered the core range for most species. This analysis was only possible for the data from Aquamaps [30] and did not include birds or corals because they did not include information on probability of occurrence.

Although our data were the most comprehensive available, they represent a relatively small fraction of overall known marine biodiversity [36], [37], which is poorly documented for many taxa. Current species distribution databases are particularly biased towards ray-finned fishes (Actinopterygii) and other vertebrates. Not surprisingly, the taxonomic makeup of species in our analysis was not proportional to the number of species within these taxonomic groups overall. Therefore, we explored the effects of weighting the number of species in each taxonomic group by their estimated proportional representation of all species within that group, excluding Actinopterygii. This approach was designed to reduce the relative importance of some classes that are poorly represented in the dataset used here because the species represented in these classes may not be reflective of their overall taxonomic group patterns (i.e. Echinodermata, Arthopoda, Mollusca) (Table S2). We then compared the top 5% by richness using this weighted approach to the equal weighting approach used in the main analyses.

To explore taxon-specific drivers of patterns in priority area identification, we conducted an additional set of analyses where we repeated our priority setting methodology for eight taxonomic groups separately (Arthropoda, Ascidiacea, Aves, Cnidaria, Echinodermata, Elasmobranchii, Mammalia, and Mollusca) to create taxon-specific priority areas. We calculated priorities for these taxa only for richness because many taxa had relatively low variation in endemism values. We then compared taxon-specific results to the cross-taxa priorities in our main analysis.

## Results and Discussion

Human impacts can disproportionately affect areas of high biodiversity [25] so spatially quantifying the degree of human impact in these areas is a key component of conservation prioritization efforts [16], [20], [21]. We used a global model of estimated human impacts (Fig. 1D) [1] to understand how human activities may be affecting marine biodiversity within Exclusive Economic Zones (EEZs) and Areas Beyond National Jurisdiction (ABNJ). Priority areas for conservation were characterized by high richness, range rarity, or proportional range rarity and relatively high or low levels of human impact. Our results highlighted not only places that are accepted priorities for marine conservation, like the Coral Triangle, but also less well-known places like the southwest Indian Ocean, western Pacific Ocean, semi-enclosed seas like the Mediterranean, Baltic and Black Seas and along eastern boundary current systems and the Antarctic and Arctic Oceans in ABNJ.

### Biodiversity patterns

**Figure 2:**
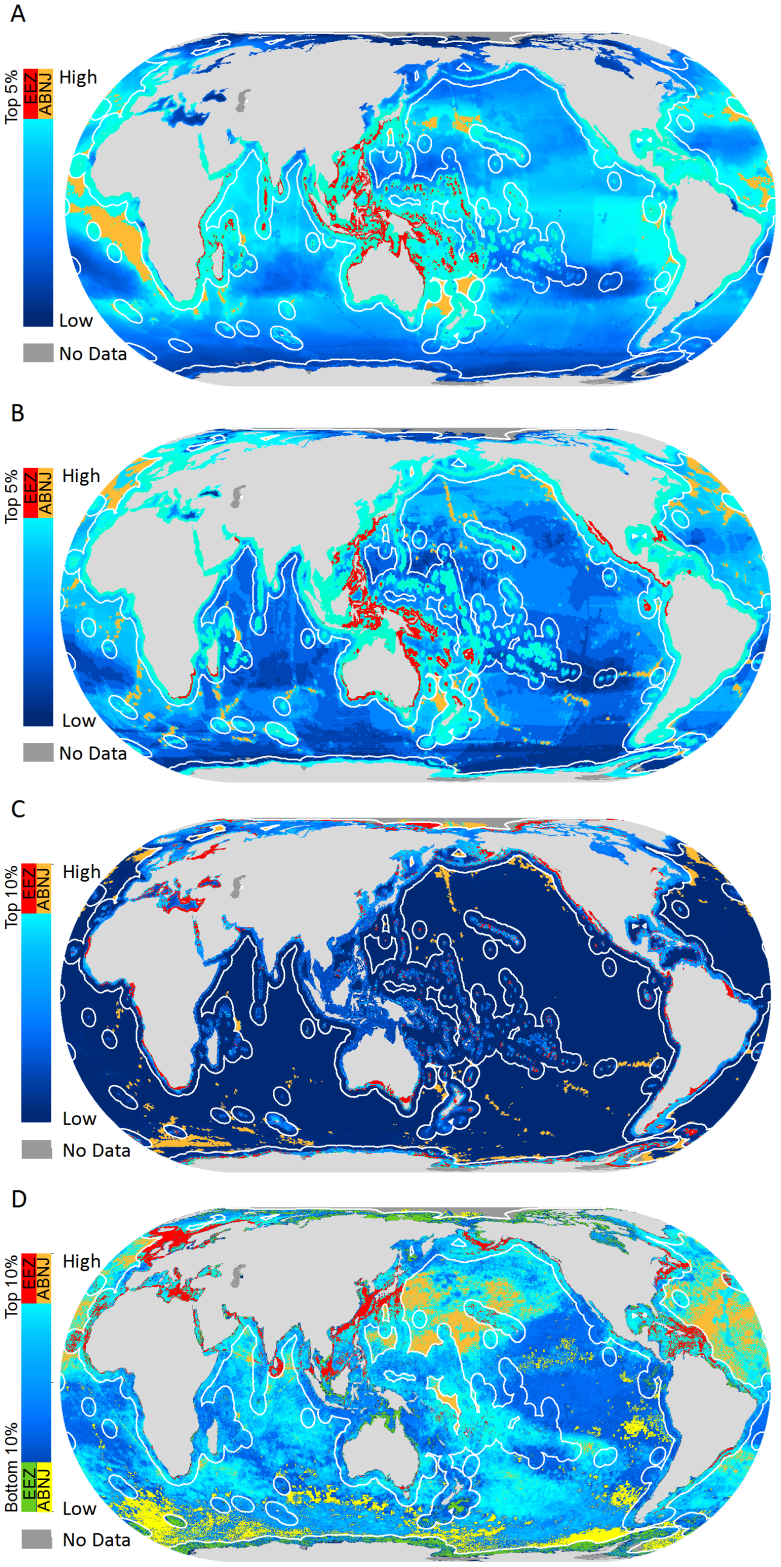
Spatial patterns for (A) species richness, (B) range rarity, and (C) proportional range rarity and (D) cumulative human impacts within EEZs and ABNJ. The highest values for all diversity measures within 5% of EEZ or ANBJ area are also shown. Due to scale, not all values may be visible. EEZ boundaries are shown in white.

Overall, biodiversity peaks within EEZs and ABNJ (Fig. 2A–B) generally followed well-documented patterns [20], [25], [38], [39], but we also identified places of high biodiversity that are less often considered, especially for range rarity and proportional range rarity, which have not been explored previously at global scales across so many taxa (Fig. 2A). To test the accuracy of the modeled species range data that we used for our analysis, we looked at the relationship between normalized richness values in our database with those in a different global database of species distributions that uses a somewhat different suite of species and a coarser resolution [25] and found a relatively strong correlation (R^2^ = 0.5812; p-value<2.2×10^−16^).

Peaks in marine species richness and endemism generally occur in the tropics, although temperate areas were also identified, particularly for proportional range rarity (Fig. 2C). We found high species richness values (range of top 5% = 1618–5099 species) in the EEZ waters of several Southeast Asian nations in the region known as the Coral Triangle. We also found higher richness in less well-known places in the Indian Ocean along the coasts of Madagascar and the Chagos, Maldives and Lakshadweep archipelagos. In contrast, higher range rarity values within EEZs (range of top 5% = 301–680) were most prevalent in the Coral Triangle, the Bahamas, and along the Pacific Central American coast (Fig. 2B). High proportional range rarity values (range of top 5% = 376–500) were found along Arctic and Antarctic coasts and within semi-enclosed seas such as the Mediterranean, Baltic, and Black Seas (Fig. 2C). There was only a 2% overlap between the two measures of endemism within EEZs, suggesting that they are largely complementary.

High values for all three metrics of biodiversity were found along coastal and shelf areas so the separation of EEZs from ABNJ not only reflected governance differences, but also facilitated identification of high biodiversity areas within ABNJ, where values are typically lower than in EEZs. Within ABNJ, richness peaked more strongly (range of top 5% = 424–4222) along eastern boundary currents dominated by the Canary and Benguela Current systems and more weakly off western boundary currents like the Gulf Stream and Kuroshio Current systems (Fig. 2A; Fig. S1). Peaks in range rarity (range of top 5% = 10–322) were concentrated along mid-oceanic ridge systems (Fig. 2B). For proportional range rarity, peaks were found on the Labrador and Newfoundland Basins, the Rockall Rise off the coasts of the United Kingdom and Ireland, and in the Arctic and Antarctic Oceans (range of top 5% = 48–3000; Fig. 1C). In ABNJ, there was a 12% overlap between the two measures of endemism.

Although our analyses highlighted many areas that have been previously identified as endemism hotspots for corals [20] and fish [40], we may have failed to identify small pockets of high endemism – for example, those known to exist around the Marquesas and Mascarenes islands [40] – because they occur at a resolution finer than our species range data. In addition, failures in identifying peaks in richness, range rarity, and proportional range rarity may have been affected by a lack of knowledge in some regions. Additional sampling may enable more places of high diversity to be identified, particularly in places where our knowledge of marine biodiversity is still growing [41]. Our estimates of diversity peaks may differ from more region-specific analyses [42] because of the inclusion of a different suite of species that includes many temperate species. Finally, we lacked data for many benthic and demersal off-shelf species so we were likely not able to identify places of high endemism in ocean trenches, canyons, or other deep-sea formations, so patterns within ABNJ in particular may change as more data for other taxa are incorporated.

### Priority areas

**Figure 3:**
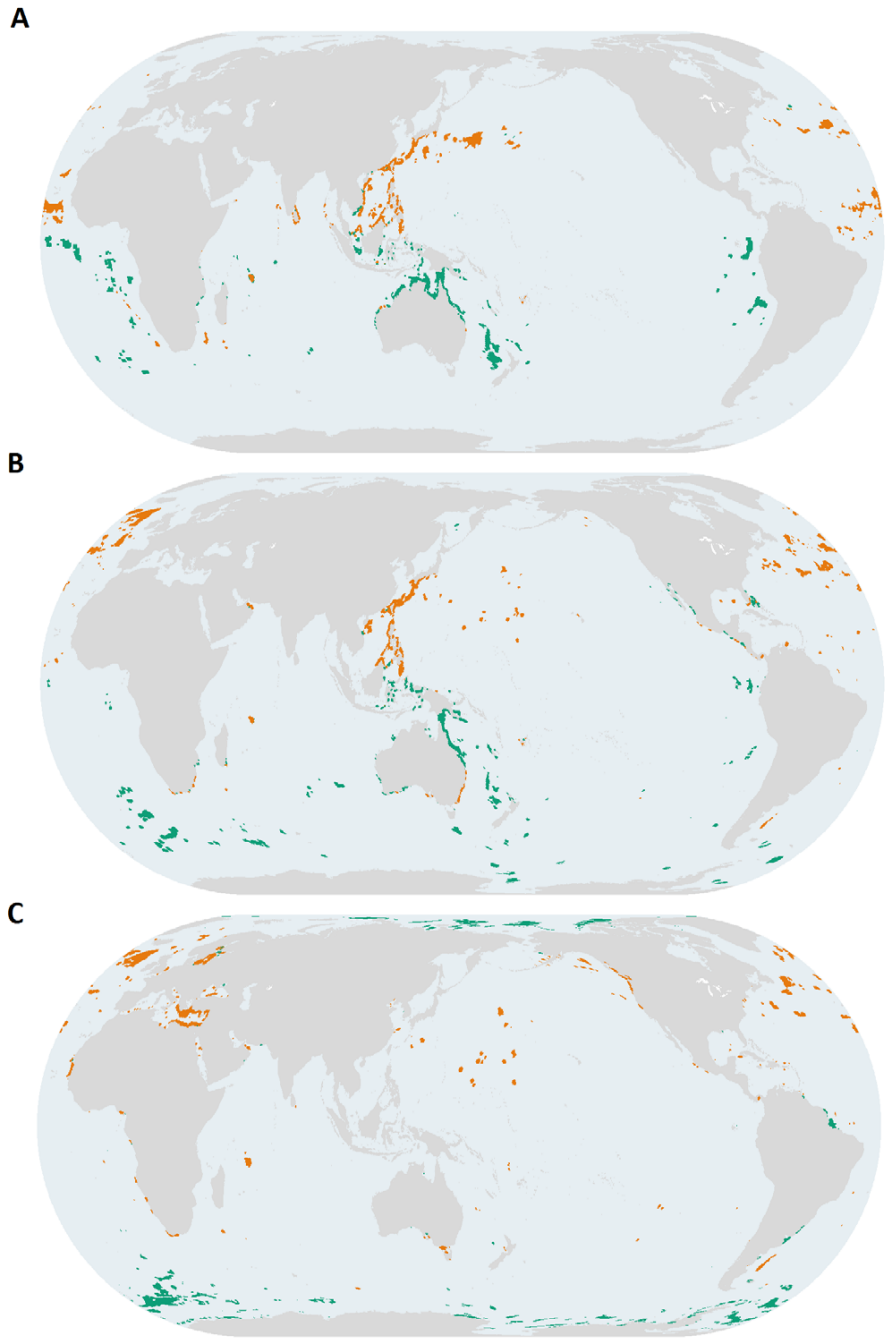
Priority areas for marine biodiversity conservation for (**A**) species richness, (**B**) range rarity, and (**C**) proportional range rarity within EEZs and ABNJ. Orange areas denote priority areas with high human impacts and green denotes areas with low human impacts. Total area of priorities is 7,233,550 km^2^ within EEZs and 9,894,560 km^2^ within ABNJ (Tables S5, S6).

The designation of priority areas was driven both by spatial patterns in diversity and human impact. When we considered the degree of human impact on these places of high biodiversity, several large areas emerged as priority areas for marine conservation (Table 1; Table S3). Areas of high biodiversity – high impact were identified around India, South Africa, Sri Lanka, Fiji, southeastern Australia, the South China Sea, the Mediterranean Sea, the Baltic Sea, and the coasts of Southeast Asia among others (Fig. 3A–C; Table S3). High biodiversity-low impact areas were identified within the Pacific EEZs of Mexico, Colombia, and Honduras as well as the Bahamas, the Galapagos (Ecuador), Madagascar, Mozambique, West Papua (Indonesia), Papua New Guinea, the north and west coasts of Australia, southern Kalimantan (Indonesia), the Solomon Islands, and in the Arctic and Antarctic Oceans (Fig. 3A–C). Some countries did not have any priority areas within their EEZs (Table S3) either because impact levels were relatively moderate or because they lacked high levels of one of the three biodiversity metrics used in the analyses.

**Table 1:** Top 20 EEZ regions by total priority area (km^2^).

EEZ region (Sovereign)	Total priority area (km2)	Percent of EEZ in priority areas	% high impact
Australia	1,094,440	16	15
Indonesia	593,450	10	28
Antarctica	502,300	6	0
Russia	367,870	5	2
Japan	358,690	9	100
Philippines	346,230	19	94
Canada	227,540	4	20
Greece	172,460	35	100
Vietnam	160,660	25	68
Papua New Guinea	152,130	6	2
China	150,170	17	96
Taiwan	127,870	37	97
Egypt	107,540	41	99
Brazil	102,950	3	0
United States	96,390	4	92
Malaysia	88,530	19	77
Turkey	85,900	34	100
Sweden	82,620	53	100
Mexico	78,690	2	44
Bahamas	78,030	13	3

Total priority area was determined through the union of priority areas based on richness, range rarity, and proportional range rarity (Fig. 3A–C). Calculations for the United States do not include EEZ regions around Alaska or Hawaii, which are calculated separately. Table S3 has statistics for all EEZ areas and further statistics on priority areas by richness, endemism and proportional range rarity. Table S4 has statistics by FAO region for ABNJ priority areas.

Australia's EEZ included the largest absolute spatial extent of priority areas, most of which were high biodiversity – low impact. Other EEZ regions that had relatively extensive high biodiversity-low impact areas included those of Indonesia, Antarctica, Russia, Canada, Papua New Guinea, Brazil, and the Bahamas (Table 1). These places represent opportunities for proactive conservation efforts to protect marine resources before they become highly impacted or to ensure that effective management is maintained where it already exists such as in Australia's Great Barrier Reef Marine Protected Area. In contrast, countries like the Philippines, Japan, and China, which had large areas of highly impacted priority areas, should be considered urgent priorities for conservation intervention (Table 1). Within EEZs, priority areas for richness and range rarity overlapped by 21% (Table S5). These areas of overlap may be particularly important for conservation efforts because they could represent areas that have high ecosystem complexity and high irreplaceability because of the levels of species richness and endemism present.

Within ABNJ, high impact priority areas for species richness were found primarily in the sub-tropics near the mid-Atlantic ridge and off the coasts of Japan and West Africa in the Canary Current system (Fig. 3A; Table S4). Low impact priority areas were found off of the coasts of southwestern Africa in the Benguela Current system, northwestern South America, and within the Arctic and Antarctic Oceans. Although high priorities for richness concentrated in the tropical latitudes, high priority areas for both measures of endemism were found in sub-tropical, temperate, and polar latitudes. When defined as discrete priority areas, between 55–65% of ABNJ priority areas also contained seamounts depending on the biodiversity metric used to define priority areas [43]. The overlap of many priority areas with seamount locations likely reflects known higher biodiversity associated with seamounts [44]. The relatively low degree of spatial concordance for priority areas for richness and either range rarity or proportional range rarity in ABNJ (11%) means that conservation measures aimed at preserving priority areas with high richness will not necessarily encompass places with high endemism (Table S6). Even though our priorities cover relatively little area relative to the overall size of ABNJ, interventions targeted at these areas (e.g. marine protected areas, fisheries management, etc.) can be considered as one component of broader conservation efforts, which could also include monitoring and regulating trade of threatened species. Recent research suggests that marine protected areas can be useful for rebuilding populations of even wide-ranging species like Atlantic cod [45].

Although we summarize priority area extent here by EEZ or ABNJ (Table 1, Table S3, Table S4), there was often considerable variability within these regions. For example, within Indonesia, priority areas were identified for West Papua and Kalimantan, but not Sulawesi. In this case and many others, impact levels varied within EEZs so places that had more moderate levels of impact were not designated as priority areas. These “moderate” impact but high biodiversity areas also have conservation value. Even though it was not the focus of this analysis, our methodology could be adapted to categorize high diversity places that fall into different categories of impact for conservation planning purposes.

To ensure that our priority area results were robust to changing the probability of occurrence thresholds, we examined the degree of overlap between the ≥0.0 threshold we used in the main analysis and two additional thresholds. As with previous studies focusing on marine mammals [35], we found that estimates of species richness were relatively robust to changing the probability of occurrence thresholds. At a >0.4 probability threshold, the top richness values within 5% of total EEZ area had an overlap of 93% with the locations identified at a ≥0.0 threshold, although overlap declined to 66% at a higher 0.8 probability threshold (Fig. S2). Range rarity and proportional range rarity values were less robust to changing the probability threshold, likely because increasing the probability threshold caused species ranges to contract idiosyncratically, which had a more direct impact on metrics of endemism (Fig. S2). Increasing probability thresholds would have identified more priority areas for richness and range rarity in the Caribbean and off the west coast of Africa (Fig. S2). For proportional range rarity, higher probability of occurrence thresholds values resulted in more priority areas along the east and west coasts of the United States, while reducing areas in the eastern Mediterranean and Baltic Seas. Many of these areas had values that were close to the top 5% of biodiversity within EEZs and would have been included if broader area thresholds were used (Fig. S3). A 10% threshold would also have increased the degree of overlap between priority areas for richness, range rarity, and proportional range rarity.

### Taxonomic considerations

Because our analyses used modeled species range data for a subset of known marine species, we conducted a series of validations and sensitivity analyses that suggested that our approach was relatively robust to different taxonomic weighting and species composition. The overall percent overlap between the highest values within 5% of EEZ area for richness with the equal species weighting and the proportional representation weighting was 70% (Fig. S5). The proportional weighting approach, which attempted to correct for taxonomic sampling biases, tended to identify larger areas within the Coral Triangle and Western Pacific regions whereas the equal weighting approach extended priority areas along the coasts of China, Japan, northern Australia, and eastern Africa (Fig. S5). The differing spatial patterns may be a result of the increased focus of the proportional weighting on a more limited set of taxa, thereby extending core areas for those taxa. Although priorities could shift with the addition of more species and classes, the priorities identified in our analysis seem relatively robust.

Overall marine conservation priorities were determined by cross-taxonomic relationships, which were dominated by ray-finned fishes (Actinopterygii; 8013 out of 12,497 species; Table S1). However, global priorities captured taxon-specific priorities relatively well for many taxa (Fig. S4; Table S7). When we compared taxon-specific richness priorities to the global cross-taxa priorities (Fig. S4), we found relatively high overlap (>60% overlap for 5 out of 8 taxa; Table S7). The most divergent taxa from global patterns were Mammalia and Aves, both of which peak in richness in high latitudes (Figs. S4F, S4H; Table S7). Global marine priority areas also had less overlap for Cnidaria (∼41%), but more than 89% overlap for Arthropoda and 93% for Mollusca (Fig.S4; Table S7), although the proportional representation of species within these taxonomic groups in our analyses was relatively low (Table S2).

Because taxa with known latitudinal counter-gradient patterns in richness like Aves and Mammalia had little overlap with global priorities (Table S7), additional areas will be needed to conserve these taxa [35], [46]. No single prioritization scheme will be optimal for all taxa so taxon-specific management goals will be better identified through focal analyses on specific taxa [26], [35], [47]. Global priority areas best serve the broadest set of taxa, but will need to be supplemented by additional areas that capture places that are important for specific taxa.

### Impacts within priority areas

To determine which types of conservation interventions may be most effective, we also assessed which impacts were driving the designation of ‘highly impacted’ in our identified priority areas. The 17 different human impact layers we used fall broadly into four general categories: climate, fishing, land-based pollution, and ocean-based pollution. Management interventions for each of these kinds of impacts may be quite different. For example, climate impacts will be most effectively managed through policy interventions to reduce the human activities causing climate change, whereas fishing impacts may be managed more locally through a combination of marine protected areas and traditional catch or effort controls. Land-based impacts can be managed through interventions like watershed management or upstream protected areas.

**Figure 4:**
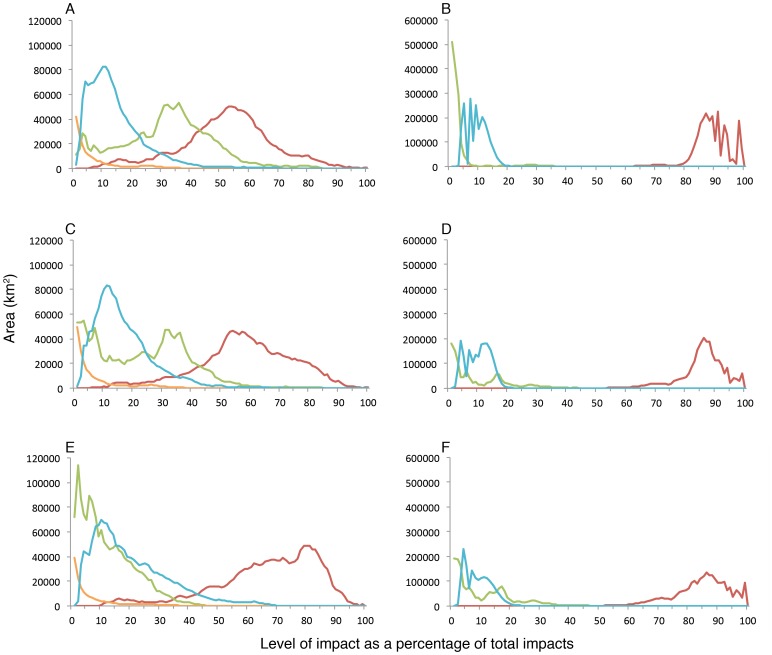
Intensity and extent of climate (red), fishing (green), ocean-based (blue) pollution, and land-based impacts (orange) as a percentage of total impacts within highly impacted priority areas. Patterns are shown for priority areas that were designated based on (**A**) richness within EEZs (**B**) and ABNJ, (**C**) range rarity within EEZ and (**D**) ABNJ, and (**E**) proportional range rarity within EEZs and (**F**) ABNJ. Zero values are not shown.

The distribution and intensity of impacts within highly impacted priority areas revealed several interesting patterns. The strong rightward skew of climate impacts (red line) in plots of the level of impact as a percentage of all impacts illustrate that they were the most intense type of impact in highly impacted priority areas, although these patterns were more pronounced in ABNJ than EEZs (Figs. 4A–F). Interestingly, impact patterns were relatively consistent in ABNJ despite the priorities covering distinctly different geographic areas (Figs. 4B, 4D, 4F). Within highly impacted priority areas in EEZs, fishing impacts were also widespread, while ocean-based pollution and land-based impacts generally had more localized effects (Figs. 4A, 4C). Patterns for proportional range rarity (Fig. 4E) differed from these overall patterns with climate impacts still having the most intense impacts, but with fishing impacts (green line) more skewed to the left, indicating less intensity in these priority areas than in those for richness (Fig. 4A) or range rarity (Fig. 4B). These results may reflect better management of fishing pressures in these areas or less intense fishing pressures. In both EEZs and ABNJ, areas in the Northern Hemisphere were generally more impacted than the Southern Hemisphere (Fig. 3). These differences may be due in part to greater climate impacts [48] and a longer history and greater intensity of fisheries exploitation in the Northern Hemisphere [49]. Although the impact data are a proxy of cumulative impacts on marine ecosystems, effects of a particular impact can vary according to species, scale, and location. Managing impacts on biodiversity will require adopting strategies to ameliorate the impacts of individual stressors on particular species or ecosystems [50]. Nonetheless, our results emphasize the critical importance of redoubling efforts towards developing policies and actions that promote sustainable fisheries management and reduce the human activities responsible for climate change. Although implementing climate policy has been challenging, a combination of management tools is still important to pursue because evidence suggests marine protected areas alone will not be able to mitigate climate change impacts [51].

### Conclusions

Our results can serve as a foundation for informing a wide range of policy and management objectives aimed at protecting marine biodiversity. Areas identified here can serve as initial components of a portfolio approach as countries move towards fulfilling Convention on Biological Diversity Aichi Target 11 for 10% of the ocean to be protected by 2020 [6]. In addition, our results can be compared with the current extent of protection to determine where gaps remain and where biodiversity may be particularly vulnerable to human impacts. Areas identified here can also be used in future analyses to determine where places with high biodiversity may also be providing important ecosystem services.

There are many other factors besides biodiversity and impact that should be assessed to meet CBD or other management targets. We specifically focused on the identifying priority areas that amounted to approximately 5% of EEZ area as stepping stones to a broader 10% goal (Fig. S3). We do not suggest that areas highlighted here should be used exclusively to fulfill a 10% CBD target. For example, we did not try to achieve “representation” of all biogeographic provinces and realms with our priority areas. Many of these additional areas would also be important for biodiversity conservation, particularly at more local scales. However, our approach could be adapted to be applied to smaller areas within an EEZ or biogeographic region to identify additional priorities.

Although our analysis focused on areas that are severely impacted or relatively unimpacted, several other variables and levels of impact may be important for marine spatial planning processes [52]. For example, even though the southern areas of the Coral Triangle were not highlighted because they have more moderate levels of impact, they may still be important for conservation efforts that consider costs and benefits of implementation. In addition, our analysis was limited to examining current stressors to marine ecosystems. We were also not able to account for synergies or negative interactions, which may have affected the range of values in our analyses. However, these interactions should not have changed our priority delineations, which were based on relative rankings. A greater understanding of synergistic or other interaction types would increase the differences between priority areas and other areas in most cases. Nonetheless, our results could be useful inputs for future cost-benefit analyses, and can be paired with scale-appropriate data on specific management options, which were not available at a global scale.

Future priority-setting and planning exercises will also need to consider socio-economic variables, governance considerations, presence of other uses or stakeholder interests, and other biological properties, some of which may be too fine-scale to be mapped regionally or globally. For example, highly productive areas for fisheries or highly productive ecosystems like salt marshes can be relatively low in species richness and endemism [38] and may not have been highlighted by our analyses. Furthermore, our analyses likely underestimated diversity in open or deep ocean ecosystems, where data are limited. Accounting for complementarity among taxa may also identify additional priority areas. In addition, variation in species-specific densities may result in areas of high biodiversity actually representing relatively marginal habitat for many species, which would reduce their conservation value [53]. Local-scale processes or values may be better captured by existing prioritization frameworks such as Key Biodiversity Areas [54] and Ecologically or Biologically Significant Areas [55].

Results from our analyses can help to guide global investment in biodiversity conservation, assist national and regional scale conservation prioritization exercises, and provide critical baselines for assessing the effectiveness of current and future management activities. Because healthy natural ecosystems are increasingly recognized as important for maintaining human well-being, identifying and conserving priority areas for marine biodiversity are critical steps towards preserving the biodiversity on which human populations depend.

## Supporting Information

Table S1 **Taxonomic groups of species in the analysis.** These taxa were used because they had publicly available data on spatial distribution. Aves (337 species) are from Birdlife International and Cnidaria (920 species) are from the Global Marine Species Assessment. All other taxonomic groups came from the AquaMaps database [30].
(DOCX)

Table S2 **Proportional weighting by taxa used for sensitivity analysis.** All estimates of total taxonomic diversity are from Bouchet *et al.* [37] except for Aves (Birdlife International), Elasmobranchii (IUCN Shark Specialist Group) and Mammalia [46].
(DOCX)

Table S3 **Total priority area (km^2^) within EEZs.** Area estimates have been rounded to the nearest 10 km. EEZ boundaries may still be in dispute. Overlap refers to areas of overlap between richness, range rarity, or proportional range rarity in any combination. Countries not listed did not have priority areas identified by the global analysis because they lacked spatially concordant high levels of diversity and high impact or low impact.
(DOCX)

Table S4 **Total priority area (km^2^) within ABNJ by FAO regions.** Area estimates have been rounded to the nearest 10 km. Overlap refers to areas of overlap between richness, range rarity or proportional range rarity in any combination.
(DOCX)

Table S5 **Area of priority areas (km^2^) within EEZs by level of impact and type of priority.** Area estimates have been rounded to the nearest 10 km.
(DOCX)

Table S6 **Area of priority areas (km^2^) within ABNJ by level of impact and type of priority.** Area estimates have been rounded to the nearest 10 km.
(DOCX)

Table S7 **Percent overlap between taxon-specific priorities and global cross-taxa priorities for species richness.**
(DOCX)

Figure S1 **Continuous values for (A) richness, (B) range rarity, (C) proportional range rarity, and (D) cumulative impact values** [**1**] **for all ocean areas.** For analytical purposes, range rarity values were multiplied by 100,000 and proportional range rarity values by 1,000 to create integer datasets.
(TIF)

Figure S2 **Changes in priority areas using different probability thresholds for (A) richness, (B) range rarity, and (C) proportional range rarity.** Priority areas differ from those in the main analysis because only data from Aquamaps were used for this analysis. Aquamaps is the only species range dataset that has probability of occurrence information. The biggest changes were in priority areas designated according to proportional range rarity. The Caribbean and off the western coast of Africa also had differences for richness and range rarity.
(TIF)

Figure S3 **Marine priorities within Exclusive Economic Zones using 10% area threshold for (A) richness, (B) range rarity, and (C) proportional range rarity.**
(TIF)

Figure S4 **Taxa-specific priorities for (A) Arthropoda, (B) Ascidiacea, (C) Cnidaria, (D) Echinodermata, (E) Elasmobranchii, (F) Mammalia, (G) Mollusca, and (H) Aves.** Orange areas are in places of high human impact and green areas are in places of low human impact.
(TIF)

Figure S5 **Comparison of equal weighting of species versus proportion weighting by representation within each taxonomic group for the top 5% of EEZ area by richness.** Overlap between the two approaches is in light green, equal weighting is in dark blue, and proportional weighting is in yellow.
(TIF)
