# Supplementary material for: Global Priorities for Marine Biodiversity Conservation
Source: PLoS One. 2014 Jan 8;9(1):e82898. doi: 10.1371/journal.pone.0082898 (PMC3885410; doi:10.1371/journal.pone.0082898)
Supplement: Table S1 — Taxonomic groups of species in the analysis. These taxa were used because they had publicly available data on spatial distribution. Aves (337 species) are from Birdlife International and Cnidaria (920 species) are from the Global Marine Species Assessment. All other taxonomic groups came from the AquaMaps database [30]. (DOCX) [file pone.0082898.s001.docx]

| **Taxonomic group** | **Number of species** |
| --- | --- |
| Acanthocephala, Entoprocta, Nemertea, Phoronida, Unassigned (1 each) | 5 |
| Annelida | 35 |
| Arthropoda | 688 |
| Brachiopoda | 11 |
| Bryozoa | 24 |
| Cephalorhyncha | 3 |
| Chaetognatha | 14 |
| Chlorophyta | 19 |
| Chordata | 9,300 |
| \| Actinopterygii \| \| --- \| \| Appendicularia \| \| Ascidiacea \| \| Aves \| \| Cephalaspidomorphi \| \| Cephalochordata \| \| Elasmobranchii \| \| Holocephali \| \| Mammalia \| \| Myxini \| \| Reptilia \| \| Sarcopterygii \| \| Thaliacea \| | \| 8,013 \| \| --- \| \| 6 \| \| 178 \| \| 337 \| \| 6 \| \| 5 \| \| 571 \| \| 25 \| \| 118 \| \| 15 \| \| 19 \| \| 1 \| \| 6 \| |
| Cnidaria | 920 |
| Ctenophora | 2 |
| Echinodermata | 67 |
| Gastrotricha | 12 |
| Mollusca | 1,298 |
| Ochrophyta | 15 |
| Porifera | 30 |
| Rhodophyta | 19 |
| Sipuncula | 35 |
| **Total** | **12,497** |
