# Supplementary material for: Global Priorities for Marine Biodiversity Conservation
Source: PLoS One. 2014 Jan 8;9(1):e82898. doi: 10.1371/journal.pone.0082898 (PMC3885410; doi:10.1371/journal.pone.0082898)
Supplement: Table S2 — Proportional weighting by taxa used for sensitivity analysis. All estimates of total taxonomic diversity are from Bouchet et al. [37] except for Aves (Birdlife International), Elasmobranchii (IUCN Shark Specialist Group) and Mammalia [46]. (DOCX) [file pone.0082898.s002.docx]

| **Taxon** | **Percent overlap with global priorities for richness** | **Percent overlap with all global priorities** |
| --- | --- | --- |
| Arthropoda | 84 | 89 |
| Ascidiacea | 58 | 60 |
| Aves | 2 | 15 |
| Cnidaria | 41 | 47 |
| Echinodermata | 67 | 70 |
| Elasmobranchii | 76 | 82 |
| Mammalia | 7.5 | 15 |
| Molluscsa | 91 | 93 |
