# Supplementary material for: Global Priorities for Marine Biodiversity Conservation
Source: PLoS One. 2014 Jan 8;9(1):e82898. doi: 10.1371/journal.pone.0082898 (PMC3885410; doi:10.1371/journal.pone.0082898)
Supplement: Table S3 — Total priority area (km2) within EEZs. Area estimates have been rounded to the nearest 10 km. EEZ boundaries may still be in dispute. Overlap refers to areas of overlap between richness, range rarity, or proportional range rarity in any combination. Countries not listed did not have priority areas identified by the global analysis because they lacked spatially concordant high levels of diversity and high impact or low impact. (DOCX) [file pone.0082898.s003.docx]

| **EEZ region (Sovereign)** | **Total priority area (km^2^)** | **Percent of EEZ in priority areas** | **Priority areas for richness (km^2^)** | **% high impact** | **Priority areas for range rarity (km^2^)** | **% high impact** | **Priority areas for proportional range rarity (km^2^)** | **% high impact** | **Priority areas (overlap) (km^2^)** | **% high impact** |
| --- | --- | --- | --- | --- | --- | --- | --- | --- | --- | --- |
| Australia | 1,094,440 | 16 | 502,300 | 4 | 232,135 | 32 | 65,570 | 85 | 294,430 | 4 |
| Indonesia | 593,450 | 10 | 271,480 | 33 | 166,560 | 11 | 0 |  | 155,410 | 38 |
| Antarctica | 502,300 | 6 | 0 |  | 0 |  | 502,300 | 0 | 0 |  |
| Russia | 367,870 | 5 | 0 |  | 0 |  | 367,870 | 2 | 0 |  |
| Japan | 358,690 | 9 | 37,380 | 100 | 73,444 | 100 | 9,840 | 100 | 238,040 | 100 |
| Philippines | 346,230 | 19 | 8,520 | 100 | 64,919 | 80 | 0 |  | 272,790 | 98 |
| Canada | 227,540 | 4 | 0 |  | 0 |  | 227,540 | 20 | 0 |  |
| Greece | 172,460 | 35 | 0 |  | 0 |  | 172,460 | 100 | 0 |  |
| Vietnam | 160,660 | 25 | 142,950 | 71 | 1,311 | 0 | 0 |  | 16,390 | 48 |
| Papua New Guinea | 152,130 | 6 | 11,800 | 0 | 64,919 | 4 | 0 |  | 75,410 | 0 |
| China | 150,170 | 17 | 35,410 | 85 | 15,738 | 96 | 25,570 | 100 | 73,440 | 100 |
| Taiwan | 127,870 | 37 | 6,560 | 100 | 22,951 | 97 | 0 |  | 98,360 | 97 |
| Egypt | 107,540 | 41 | 0 |  | 0 |  | 107,540 | 99 | 0 |  |
| Brazil | 102,950 | 3 | 0 |  | 0 |  | 102,950 | 0 | 0 |  |
| United States | 96,390 | 4 | 0 |  | 28,853 | 84 | 67,540 | 96 | 660 | 0 |
| Malaysia | 88,530 | 19 | 43,280 | 64 | 4,590 | 29 | 0 |  | 40,660 | 97 |
| Turkey | 85,900 | 34 | 0 |  | 0 |  | 85,900 | 100 | 0 |  |
| Sweden | 82,620 | 53 | 0 |  | 0 |  | 82,620 | 100 | 0 |  |
| Mexico | 78,690 | 2 | 0 |  | 48,525 | 11 | 13,110 | 100 | 17,710 | 96 |
| Bahamas | 78,030 | 13 | 0 |  | 78,034 | 8 | 0 |  | 3,280 | 0 |
| Libya | 76,720 | 22 | 0 |  | 0 |  | 76,720 | 100 | 0 |  |
| Spratly Islands (Disputed) | 76,070 | 17 | 76,070 | 100 | 0 |  | 0 |  | 0 |  |
| South Georgia and the South Sandwich Islands (UK) | 72,130 | 5 | 0 |  | 0 |  | 72,130 | 0 | 0 |  |
| New Caledonia | 70,820 | 5 | 9,180 | 0 | 41,312 | 0 | 0 |  | 20,330 | 0 |
| South Africa | 64,260 | 6 | 0 |  | 27,541 | 86 | 17,710 | 100 | 19,020 | 100 |
| Sri Lanka | 63,610 | 12 | 59,020 | 98 | 0 |  | 3,930 | 100 | 660 | 100 |
| Alaska (US) | 59,670 | 2 | 0 |  | 0 |  | 59,670 | 57 | 0 |  |
| Galapagos Islands (Ecuador) | 56,390 | 7 | 0 |  | 55,739 | 0 | 0 |  | 660 | 0 |
| Paracel Islands (Disputed) | 47,210 | 16 | 3,930 | 100 | 7,213 | 100 | 0 |  | 36,070 | 100 |
| Mauritania | 43,940 | 28 | 0 |  | 0 |  | 43,940 | 94 | 0 |  |
| Conflict Zone (Disputed) | 42,620 | 58 | 0 |  | 26,886 | 100 | 0 |  | 15,740 | 100 |
| Oman | 40,660 | 8 | 0 |  | 17,705 | 81 | 3,280 | 60 | 20,980 | 94 |
| New Zealand | 38,030 | 1 | 0 |  | 31,476 | 0 | 6,560 | 100 | 0 |  |
| Finland | 34,750 | 44 | 0 |  | 0 |  | 34,750 | 62 | 0 |  |
| Argentina | 34,100 | 3 | 0 |  | 0 |  | 34,100 | 6 | 0 |  |
| India | 33,440 | 2 | 33,440 | 100 | 0 |  | 0 |  | 0 |  |
| Greenland (Denmark) | 32,130 | 1 | 0 |  | 0 |  | 32,130 | 0 | 0 |  |
| Mozambique | 30,820 | 5 | 19,670 | 0 | 9,836 | 0 | 0 |  | 1,310 | 0 |
| Poland | 26,890 | 84 | 0 |  | 0 |  | 26,890 | 100 | 0 |  |
| United Kingdom | 26,230 | 3 | 0 |  | 0 |  | 26,230 | 100 | 0 |  |
| Bouvet Island (Norway) | 24,260 | 6 | 0 |  | 0 |  | 24,260 | 0 | 0 |  |
| Madagascar | 22,950 | 2 | 9,180 | 0 | 11,803 | 39 | 0 |  | 2,620 | 75 |
| Italy | 22,300 | 4 | 0 |  | 0 |  | 22,300 | 100 | 0 |  |
| Venezuela | 20,330 | 4 | 0 |  | 7,869 | 100 | 9,180 | 36 | 3,280 | 100 |
| Latvia | 19,670 | 68 | 0 |  | 0 |  | 19,670 | 97 | 0 |  |
| Andaman and Nicobar Islands (India) | 19,020 | 3 | 19,020 | 100 | 0 |  | 0 |  | 0 |  |
| Algeria | 19,020 | 15 | 0 |  | 0 |  | 19,020 | 100 | 0 |  |
| Fiji | 18,360 | 1 | 660 | 100 | 7,213 | 55 | 1,310 | 100 | 9,180 | 100 |
| British Indian Ocean Territory (UK) | 18,360 | 3 | 18,360 | 0 | 0 |  | 0 |  | 0 |  |
| Cyprus | 17,710 | 18 | 0 |  | 0 |  | 17,710 | 100 | 0 |  |
| Ukraine | 17,050 | 13 | 0 |  | 0 |  | 17,050 | 73 | 0 |  |
| Namibia | 16,390 | 3 | 0 |  | 0 |  | 16,390 | 100 | 0 |  |
| Faeroe Islands (Denmark) | 16,390 | 6 | 0 |  | 0 |  | 16,390 | 100 | 0 |  |
| Panama | 15,740 | 5 | 0 |  | 3,934 | 100 | 0 |  | 11,800 | 100 |
| Saudi Arabia | 15,740 | 7 | 0 |  | 0 |  | 15,740 | 100 | 0 |  |
| Nicaragua | 15,080 | 10 | 0 |  | 15,082 | 22 | 0 |  | 0 |  |
| Western Sahara | 14,430 | 5 | 0 |  | 0 |  | 14,430 | 100 | 0 |  |
| Estonia | 13,770 | 38 | 0 |  | 0 |  | 13,770 | 86 | 0 |  |
| Cuba | 13,770 | 4 | 0 |  | 0 |  | 13,770 | 100 | 0 |  |
| Senegal | 13,110 | 8 | 0 |  | 0 |  | 13,110 | 100 | 0 |  |
| Puerto Rico and Virgin Islands (US) | 13,110 | 6 | 0 |  | 0 |  | 13,110 | 100 | 0 |  |
| El Salvador | 12,460 | 13 | 0 |  | 12,459 | 100 | 0 |  | 660 | 0 |
| Nigeria | 11,800 | 6 | 0 |  | 0 |  | 11,800 | 100 | 0 |  |
| Angola | 11,800 | 2 | 0 |  | 0 |  | 11,800 | 78 | 0 |  |
| Netherlands Antilles (Netherlands) | 11,150 | 16 | 0 |  | 9,836 | 100 | 0 |  | 1,310 | 100 |
| Israel | 11,150 | 40 | 0 |  | 0 |  | 11,150 | 100 | 0 |  |
| Lebanon | 10,490 | 54 | 0 |  | 0 |  | 10,490 | 100 | 0 |  |
| Seychelles | 10,490 | 1 | 10,490 | 0 | 0 |  | 0 |  | 0 |  |
| Uruguay | 9,840 | 7 | 0 |  | 0 |  | 9,840 | 0 | 0 |  |
| Denmark | 9,180 | 100 | 0 |  | 0 |  | 9,180 | 100 | 0 |  |
| Guatemala | 9,180 | 8 | 0 |  | 9,180 | 36 | 0 |  | 0 |  |
| Falkland Islands | 8,520 | 2 | 0 |  | 0 |  | 8,520 | 0 | 0 |  |
| Syria | 8,520 | 84 | 0 |  | 0 |  | 8,520 | 100 | 0 |  |
| République du Congo | 7,210 | 18 | 0 |  | 0 |  | 7,210 | 100 | 0 |  |
| Croatia | 7,210 | 13 | 0 |  | 0 |  | 7,210 | 100 | 0 |  |
| Ecuador | 6,560 | 3 | 0 |  | 6,557 | 0 | 0 |  | 0 |  |
| Iran | 6,560 | 3 | 0 |  | 656 | 100 | 2,620 | 100 | 3,280 | 100 |
| Dominican Republic | 5,900 | 2 | 0 |  | 0 |  | 5,900 | 100 | 0 |  |
| French Guiana | 5,900 | 4 | 0 |  | 0 |  | 5,900 | 11 | 0 |  |
| Romania | 5,900 | 20 | 0 |  | 0 |  | 5,900 | 100 | 0 |  |
| Micronesia | 5,250 | 0 | 5,250 | 0 | 0 |  | 0 |  | 0 |  |
| Yemen | 5,250 | 1 | 5,250 | 100 | 0 |  | 0 |  | 0 |  |
| Pakistan | 5,250 | 2 | 0 |  | 0 |  | 5,250 | 0 | 0 |  |
| Australia - East Timor (Joint Development) | 5,250 | 15 | 5,250 | 0 | 0 |  | 0 |  | 0 |  |
| Hawaii (US) | 4,590 | 0 | 0 |  | 4,590 | 100 | 0 |  | 0 |  |
| Iceland | 4,590 | 1 | 0 |  | 0 |  | 4,590 | 100 | 0 |  |
| Tanzania | 4,590 | 2 | 4,590 | 0 | 0 |  | 0 |  | 0 |  |
| Equatorial Guinea | 3,930 | 1 | 0 |  | 0 |  | 3,930 | 100 | 0 |  |
| Costa Rica | 3,930 | 1 | 0 |  | 3,934 | 100 | 0 |  | 0 |  |
| Netherlands | 3,930 | 6 | 0 |  | 0 |  | 3,930 | 100 | 0 |  |
| Germany | 3,930 | 7 | 0 |  | 0 |  | 3,930 | 50 | 0 |  |
| Jamaica | 3,280 | 1 | 0 |  | 0 |  | 3,280 | 100 | 0 |  |
| Norway | 3,280 | 0 | 0 |  | 0 |  | 3,280 | 100 | 0 |  |
| Sudan | 3,280 | 5 | 0 |  | 0 |  | 3,280 | 100 | 0 |  |
| Spain | 3,280 | 1 | 0 |  | 0 |  | 3,280 | 100 | 0 |  |
| Australia - Papua New Guinea | 3,280 | 88 | 0 |  | 0 |  | 0 |  | 3,280 | 0 |
| Mauritius | 2,620 | 0 | 2,620 | 0 | 0 |  | 0 |  | 0 |  |
| Lithuania | 2,620 | 43 | 0 |  | 0 |  | 2,620 | 100 | 0 |  |
| Ivory Coast | 2,620 | 2 | 0 |  | 0 |  | 2,620 | 100 | 0 |  |
| Japan - Korea | 2,620 | 3 | 0 |  | 2,623 | 100 | 0 |  | 0 |  |
| France | 2,620 | 1 | 0 |  | 0 |  | 2,620 | 100 | 0 |  |
| Maldives | 2,620 | 0 | 2,620 | 100 | 0 |  | 0 |  | 0 |  |
| Solomon Islands | 2,620 | 0 | 660 | 100 | 0 |  | 1,970 | 100 | 0 |  |
| Tunisia | 2,620 | 3 | 0 |  | 0 |  | 2,620 | 100 | 0 |  |
| Serbia-Montenegro | 2,620 | 35 | 0 |  | 0 |  | 2,620 | 100 | 0 |  |
| Norfolk Island | 1,970 | 0 | 0 |  | 1,967 | 0 | 0 |  | 0 |  |
| Kenya | 1,970 | 2 | 1,970 | 100 | 0 |  | 0 |  | 0 |  |
| Bulgaria | 1,970 | 6 | 0 |  | 0 |  | 1,970 | 100 | 0 |  |
| Georgia | 1,970 | 9 | 0 |  | 0 |  | 1,970 | 100 | 0 |  |
| Anguilla | 1,970 | 2 | 0 |  | 0 |  | 1,970 | 100 | 0 |  |
| United Arab Emirates | 1,970 | 4 | 0 |  | 1,967 | 100 | 0 |  | 0 |  |
| Cameroon | 1,970 | 13 | 0 |  | 0 |  | 1,970 | 33 | 0 |  |
| Malta | 1,310 | 2 | 0 |  | 0 |  | 1,310 | 100 | 0 |  |
| Guyana | 1,310 | 1 | 0 |  | 0 |  | 1,310 | 0 | 0 |  |
| Belgium | 1,310 | 38 | 0 |  | 0 |  | 1,310 | 100 | 0 |  |
| Chile | 1,310 | 0 | 0 |  | 0 |  | 1,310 | 100 | 0 |  |
| Albania | 660 | 6 | 0 |  | 0 |  | 660 | 100 | 0 |  |
| British Virgin Islands (UK) | 660 | 1 | 0 |  | 0 |  | 660 | 100 | 0 |  |
| South Korea | 660 | 0 | 0 |  | 0 |  | 660 | 0 | 0 |  |
| Trinidad and Tobago | 660 | 1 | 0 |  | 0 |  | 660 | 100 | 0 |  |
| Colombia | 660 | 0 | 0 |  | 656 | 0 | 0 |  | 0 |  |
