# Supplementary material for: Global Priorities for Marine Biodiversity Conservation
Source: PLoS One. 2014 Jan 8;9(1):e82898. doi: 10.1371/journal.pone.0082898 (PMC3885410; doi:10.1371/journal.pone.0082898)
Supplement: Table S4 — Total priority area (km2) within ABNJ by FAO regions. Area estimates have been rounded to the nearest 10 km. Overlap refers to areas of overlap between richness, range rarity or proportional range rarity in any combination. (DOCX) [file pone.0082898.s004.docx]

| **FAO Area** | **Percent of FAO region in priority areas** | **Total priority area (km^2^)** | **Priority areas for richness (km^2^)** | **% high impact** | **Priority areas for endemism (km^2^)** | **% high impact** | **Priority areas for normalized endemism (km^2^)** | **% high impact** | **Priority areas (overlap) (km^2^)** | **% high impact** |
| --- | --- | --- | --- | --- | --- | --- | --- | --- | --- | --- |
| Atlantic, Antarctic | 21 | 1,596,740 | 0 |  | 150,820 | 0 | 1,203,950 | 0 | 241,970 | 0 |
| Atlantic, Northeast | 29 | 1,376,410 | 16,390 | 100 | 409,190 | 100 | 280,000 | 100 | 670,830 | 100 |
| Atlantic, Eastern Central | 14 | 1,319,360 | 1,070,840 | 68 | 26,230 | 100 | 55,080 | 100 | 167,220 | 85 |
| Atlantic, Southeast | 6 | 890,500 | 413,780 | 14 | 293,120 | 0 | 9,180 | 71 | 174,430 | 1 |
| Pacific, Northwest | 8 | 793,450 | 507,550 | 98 | 67,540 | 83 | 72,790 | 100 | 145,580 | 100 |
| Pacific, Southwest | 3 | 535,090 | 225,580 | 1 | 76,720 | 0 | 9,180 | 100 | 223,610 | 4 |
| Pacific, Southeast | 2 | 513,450 | 335,090 | 0 | 58,360 | 0 | 24,920 | 100 | 95,080 | 0 |
| Atlantic, Northwest | 17 | 449,840 | 6,560 | 30 | 116,720 | 100 | 110,820 | 100 | 215,740 | 100 |
| Pacific, Antarctic | 5 | 340,990 | 0 |  | 127,210 | 0 | 169,840 | 0 | 43,940 | 0 |
| Indian Ocean, Antarctic and Southern | 4 | 325,250 | 0 |  | 188,200 | 0 | 127,870 | 2 | 9,180 | 0 |
| Atlantic, Southwest | 2 | 274,100 | 129,180 | 74 | 40,000 | 28 | 34,750 | 100 | 70,160 | 79 |
| Arctic Sea | 7 | 198,690 | 0 |  | 0 |  | 198,690 | 0 | 0 |  |
| Indian Ocean, Western | 1 | 173,770 | 71,480 | 82 | 5,250 | 0 | 22,300 | 100 | 74,760 | 100 |
| Indian Ocean, Eastern | 1 | 172,460 | 15,740 | 0 | 129,180 | 0 | 14,430 | 77 | 13,110 | 0 |
| Pacific, Western Central | 2 | 158,690 | 3,280 | 0 | 0 |  | 83,280 | 100 | 72,130 | 100 |
| Atlantic, Western Central | 2 | 125,900 | 29,510 | 100 | 69,510 | 100 | 3,280 | 100 | 23,610 | 100 |
| Pacific, Northeast | 2 | 85,250 | 0 |  | 0 |  | 74,760 | 100 | 10,490 | 100 |
| Pacific, Eastern Central | 0 | 8,520 | 0 |  | 3,930 | 0 | 4,590 | 100 | 0 |  |
| Mediterranean and Black Sea | 0 | 0 | 0 |  | 0 |  | 0 |  | 0 |  |
