# Supplementary material for: Global Priorities for Marine Biodiversity Conservation
Source: PLoS One. 2014 Jan 8;9(1):e82898. doi: 10.1371/journal.pone.0082898 (PMC3885410; doi:10.1371/journal.pone.0082898)
Supplement: Table S5 — Area of priority areas (km2) within EEZs by level of impact and type of priority. Area estimates have been rounded to the nearest 10 km. (DOCX) [file pone.0082898.s005.docx]

|  | **Richness (km^2^)** | **Range rarity**  **(km^2^)** | | **Proportional range rarity (km^2^)** | **Overlap between richness and range rarity or proportional range rarity (km^2^)** | **% Overlap between richness and range rarity or proportional range rarity** | **Overlap between range rarity and proportional range rarity (km^2^)** | **% Overlap between range rarity and proportional range rarity** | **Total (km^2^)** |
| --- | --- | --- | --- | --- | --- | --- | --- | --- | --- |
| **Low impact** | 906,900 | 893,130 | 1,468,870 | | 552,140 | 14 | 660 | 0 | 3,821,700 |
| **High impact** | 539,020 | 457,710 | 1,386,250 | | 939,690 | 28 | 89,180 | 5 | 3,411,850 |
| **Total** | 1,445,920 | 1,350,840 | 2,855,120 | | 1,491,830 | 21 | 89,840 | 2 | 7,233,550 |
