# Supplementary material for: Global Priorities for Marine Biodiversity Conservation
Source: PLoS One. 2014 Jan 8;9(1):e82898. doi: 10.1371/journal.pone.0082898 (PMC3885410; doi:10.1371/journal.pone.0082898)
Supplement: Table S6 — Area of priority areas (km2) within ABNJ by level of impact and type of priority. Area estimates have been rounded to the nearest 10 km. (DOCX) [file pone.0082898.s006.docx]

|  | **Richness (km^2^)** | **Endemism (km^2^)** | **Normalized endemism (km^2^)** | **Overlap between richness and endemism or normalized endemism (km^2^)** | **% Overlap between richness and endemism or normalized endemism** | **Overlap between endemism and normalized endemism (km^2^)** | **% Overlap between endemism and normalized endemism** | **Total (km^2^)** |
| --- | --- | --- | --- | --- | --- | --- | --- | --- |
| **Low impact** | 1,511,500 | 1,201,980 | 1,702,980 | 500,990 | 9 | 375,740 | 7 | 5,293,190 |
| **High impact** | 1,536,420 | 700,340 | 907,550 | 599,340 | 13 | 857,720 | 19 | 4,601,370 |
| **Total** | 3,047,920 | 1,902,320 | 2,610,530 | 1,100,330 | 11 | 1,233,460 | 12 | 9,894,560 |
