# Supplementary figures and images for: Global Priorities for Marine Biodiversity Conservation
Source: PLoS One. 2014 Jan 8;9(1):e82898. doi: 10.1371/journal.pone.0082898 (PMC3885410; doi:10.1371/journal.pone.0082898)

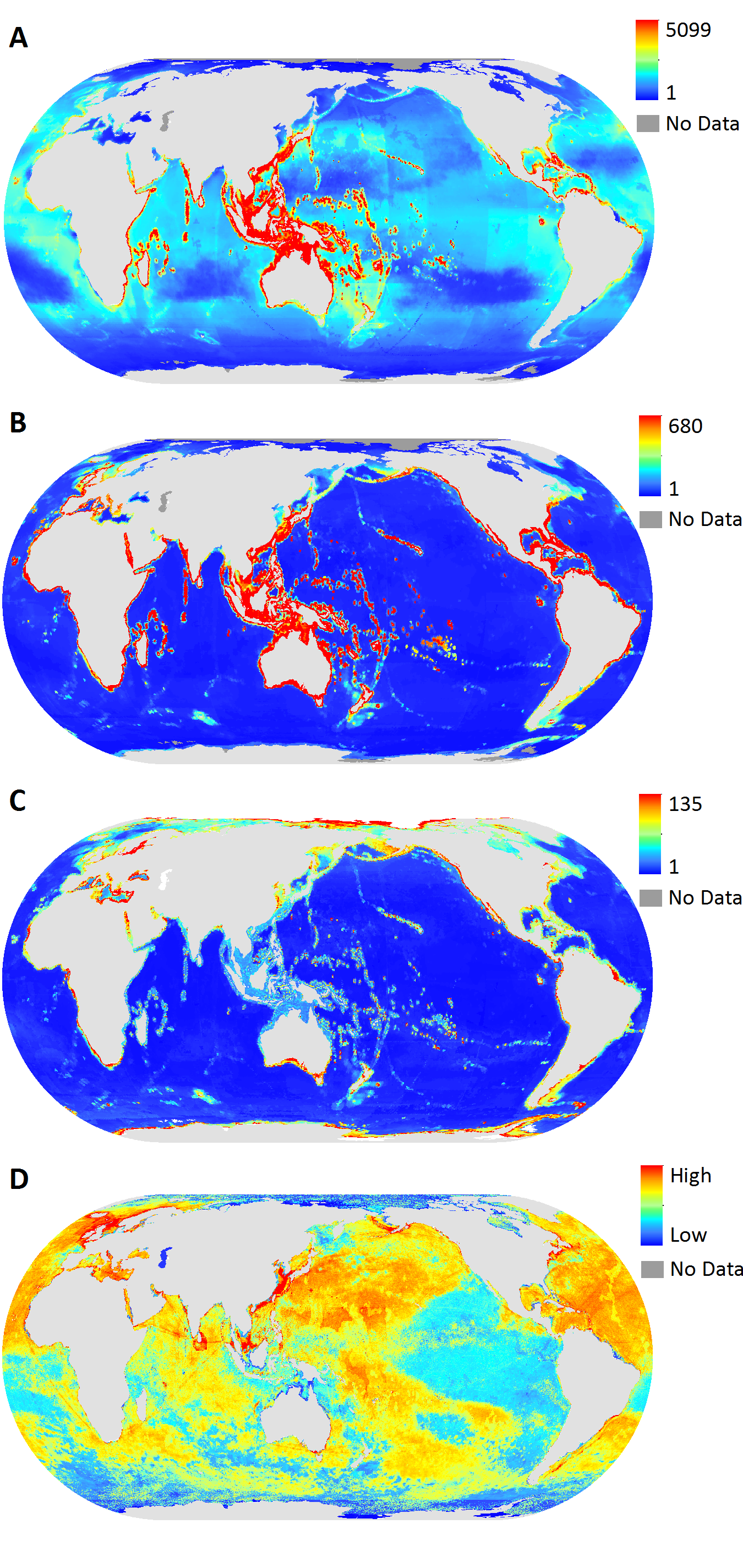

Supplement: Figure S1 — Continuous values for (A) richness, (B) range rarity, (C) proportional range rarity, and (D) cumulative impact values [1] for all ocean areas. For analytical purposes, range rarity values were multiplied by 100,000 and proportional range rarity values by 1,000 to create integer datasets. (TIF) [file pone.0082898.s008.tif]

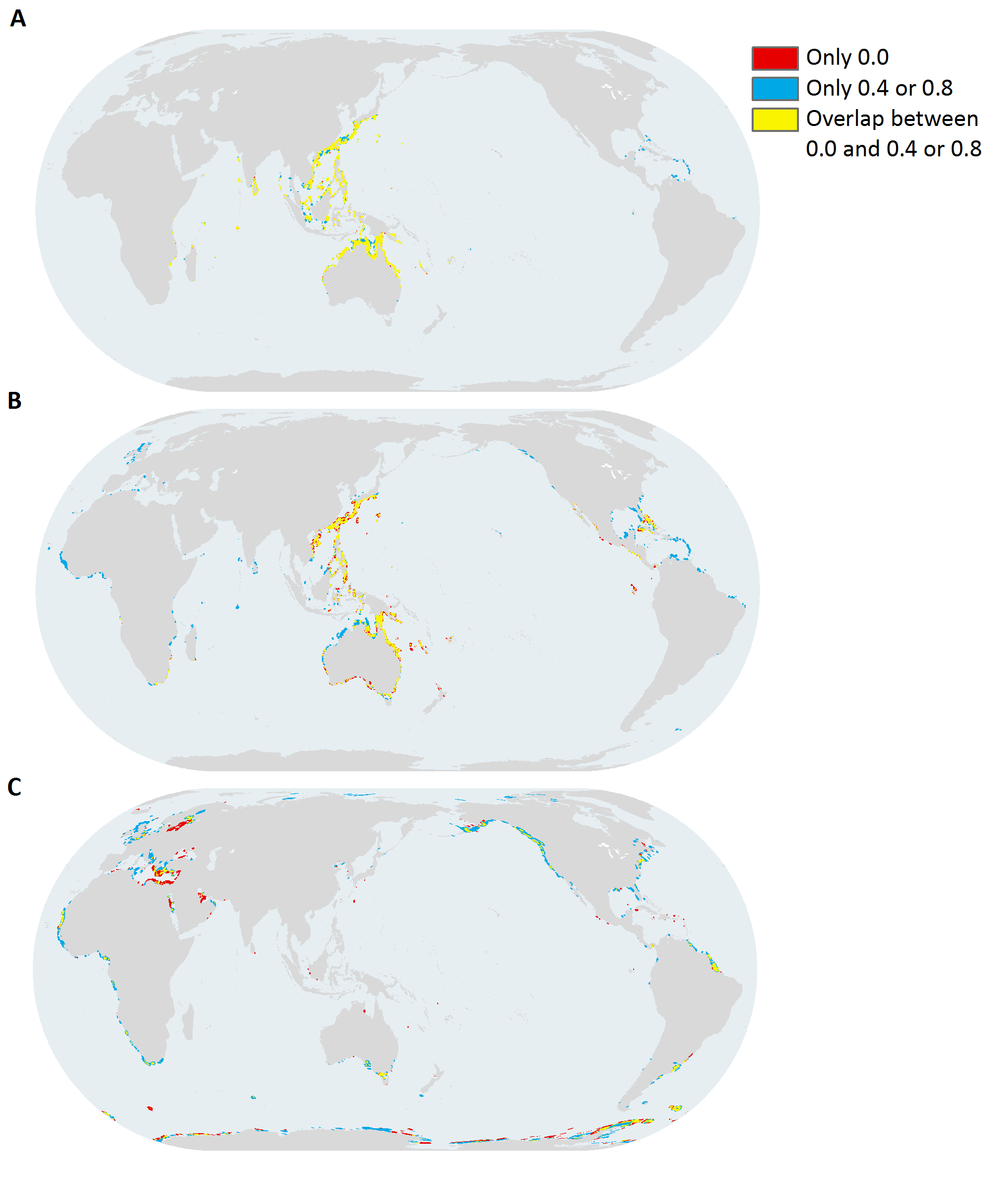

Supplement: Figure S2 — Changes in priority areas using different probability thresholds for (A) richness, (B) range rarity, and (C) proportional range rarity. Priority areas differ from those in the main analysis because only data from Aquamaps were used for this analysis. Aquamaps is the only species range dataset that has probability of occurrence information. The biggest changes were in priority areas designated according to proportional range rarity. The Caribbean and off the western coast of Africa also had differences for richness and range rarity. (TIF) [file pone.0082898.s009.tif]

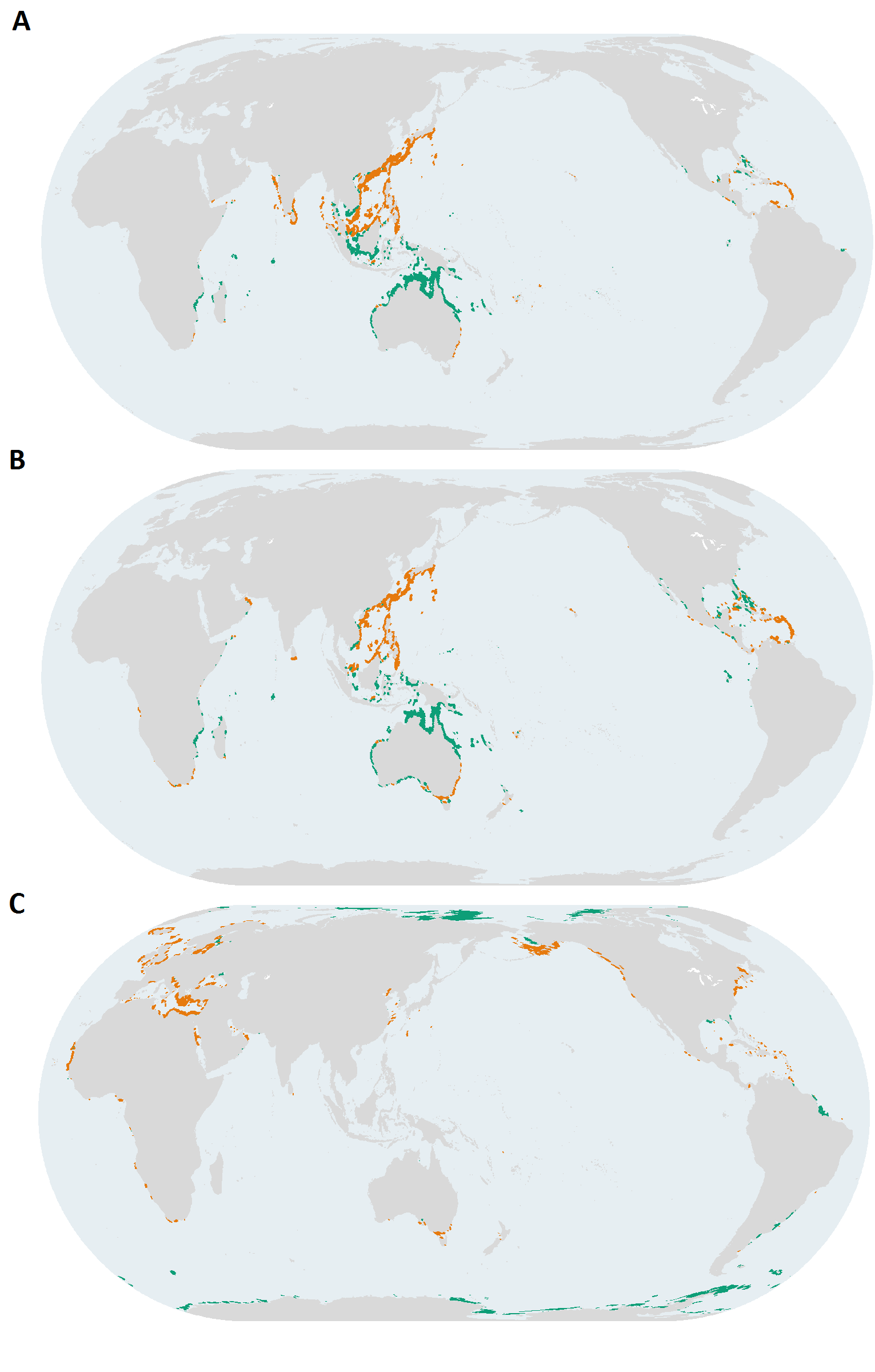

Supplement: Figure S3 — Marine priorities within Exclusive Economic Zones using 10% area threshold for (A) richness, (B) range rarity, and (C) proportional range rarity. (TIF) [file pone.0082898.s010.tif]

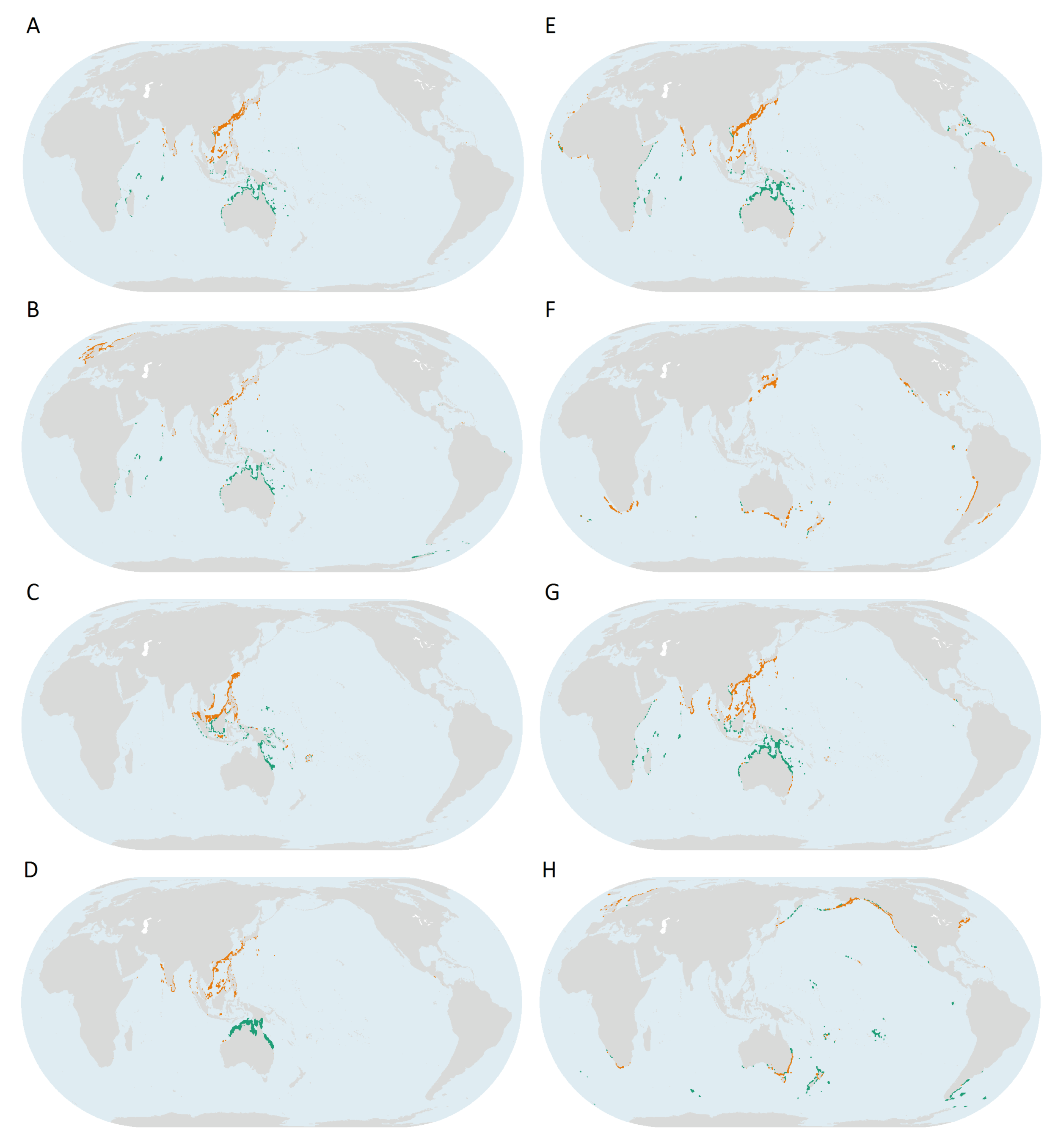

Supplement: Figure S4 — Taxa-specific priorities for (A) Arthropoda, (B) Ascidiacea, (C) Cnidaria, (D) Echinodermata, (E) Elasmobranchii, (F) Mammalia, (G) Mollusca, and (H) Aves. Orange areas are in places of high human impact and green areas are in places of low human impact. (TIF) [file pone.0082898.s011.tif]

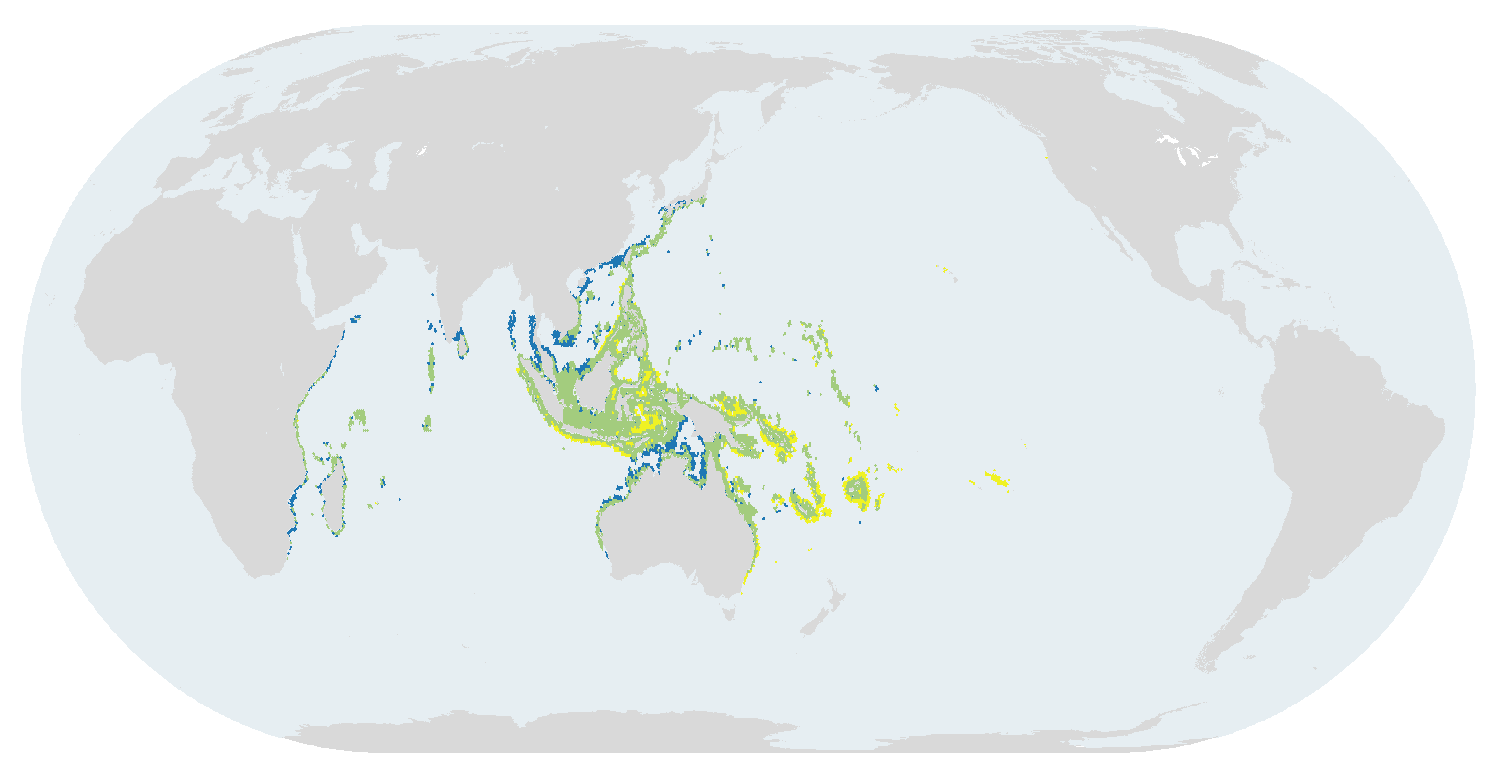

Supplement: Figure S5 — Comparison of equal weighting of species versus proportion weighting by representation within each taxonomic group for the top 5% of EEZ area by richness. Overlap between the two approaches is in light green, equal weighting is in dark blue, and proportional weighting is in yellow. (TIF) [file pone.0082898.s012.tif]
